# A time-series analysis of short-term ambient ozone exposure and hospitalizations from acute myocardial infarction in Henan, China

**DOI:** 10.1007/s11356-023-28456-2

**Published:** 2023-07-28

**Authors:** Yulong Wei, Lin Fei, Yongbin Wang, Min Zhang, Zhigang Chen, Huige Guo, Shiqi Ge, Sen Zhu, Pingshuan Dong, Kan Yang, Na Xie, Guoan Zhao

**Affiliations:** 1 Department of Cardiology, The First Affiliated Hospital of Xinxiang Medical University, Weihui, 453100 China; 2 Life Science Research Center, The First Affiliated Hospital of Xinxiang Medical University, Weihui, 453100 China; 3 Department of Epidemiology and Health Statistics, School of Public Health, Xinxiang Medical University, Xinxiang, 453003 China; 4 School of Cardiovascular and Metabolic Medicine & Sciences, King’s College London British Heart Foundation Centre of Research Excellence, London, SE5 9NU UK; 5 Department of Cardiology, The First Affiliated Hospital of Henan University of Science and Technology, Luoyang, 471003 China; 6 Department of Cardiovascular Surgery, Nanyang Affiliated Hospital of Zhengzhou University, Nanyang Central Hospital, Nanyang, 473009 China; 7 The Cardiology Department of the Third Affiliated Hospital of Xinxiang Medical University, Xinxiang, 453003 China

**Keywords:** Acute myocardial infarction, Ozone, Air pollution, Distributed lag nonlinear model, Time-series analysis

## Abstract

Epidemiological studies in recent years have identified an association between exposure to air pollutants and acute myocardial infarction (AMI); however, the association between short-term ozone (O_3_) exposure and AMI hospitalization remains unclear, particularly in developing countries. Therefore, this study collected information on 24,489 AMI patients, including daily air pollutant and meteorological data in Henan, China, between 2016 and 2021. A distributed lagged nonlinear model combined with a Poisson regression model was used to estimate the nonlinear lagged effect of O_3_ on AMI hospitalizations. We also quantified the effects of O_3_ on the number of AMI hospitalizations, hospitalization days, and hospitalization costs. The results showed that single- and dual-pollution models of O_3_ at lag0, lag1, and lag (01–07) were risk factors for AMI hospitalizations, with the most significant effect at lag03 (RR = 1.132, 95% CI:1.083–1.182). Further studies showed that males, younger people (15–64 years), warm seasons, and long sunshine duration were more susceptible to O_3_. Hospitalizations attributable to O_3_ during the study period accounted for 11.66% of the total hospitalizations, corresponding to 2856 patients, 33,492 hospital days, and 90 million RMB. Maintaining O_3_ at 10–130 µg/m^3^ can prevent hundreds of AMI hospitalizations and save millions of RMB per year in Henan, China. In conclusion, we found that short-term exposure to O_3_ was significantly associated with an increased risk of hospitalization for AMI in Henan, China, and that further reductions in ambient O_3_ levels may have substantial health and economic benefits for patients and local healthcare facilities.

**Graphical Abstract:**

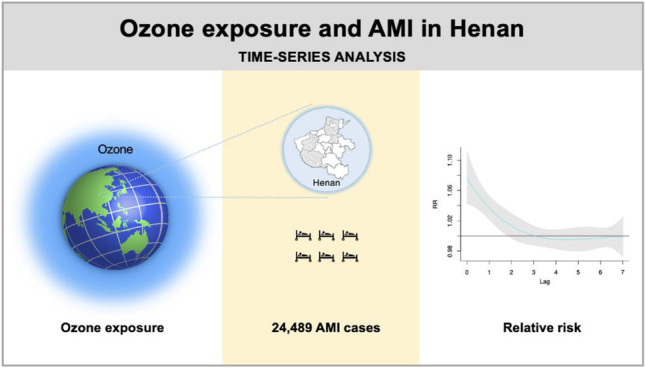

**Supplementary Information:**

The online version contains supplementary material available at 10.1007/s11356-023-28456-2.

## Introduction

Acute myocardial infarction (AMI) is known for its high morbidity and mortality (Hoole and Bambrough 2020, Reed et al. 2017); in China, AMI mortality has more than doubled in the past 20 years (Yang et al. 2013). The World Bank estimates that the number of patients with AMI in China will reach 23 million by 2030 (Bank. 2013). There is increasing evidence that exposure to ambient air pollutants is associated with an increased incidence of AMI (Chen et al. 2018; Ishii et al. 2021; Liu et al. 2021). However, most of these are studies on particulate matter and AMI incidence, with few involving ozone (O_3_), particularly in developing countries, and inconsistent outcomes. O_3_ is a secondary pollutant with strong oxidizing properties (Liu et al. 2022). The consequent temperature increase may lead to increased O_3_ concentrations in the future owing to the increased greenhouse gas emissions in the environment, accelerating global warming (Pu et al. 2017). Wang et al. (2017) reported that this process may further accelerate with the rapid consumption of fossil fuels in China. The World Health Organization (WHO) updated the WHO Global Air Quality Guidelines in 2021 to address the health hazards of O_3_ exposure by adding a new target of ambient seasonal O_3_ concentrations of less than 60 µg/m^3^ (Burki 2021); thus, ambient O_3_ pollution is a serious global public health problem.

In environmental ecology studies, short-term exposure to O_3_ increases the risk of cardiovascular disease, stroke, respiratory disease onset, and mortality (EPA US 2020). Recently, large cohort studies in the USA and Canada have shown that long-term exposure to O_3_ may be associated with mortality from cardiovascular diseases (Cakmak et al. 2016; Lim et al. 2019). In addition, a meta-study reported evidence of increased all-cause and cardiovascular mortalities associated with exposure to high O_3_ levels (Stafoggia et al. 2010). However, most of these studies were conducted at long-term O_3_ exposure levels and in developed countries where O_3_ concentrations are mostly below 60 µg/m^3^ (the ambient air quality standard for O_3_ in China is 160 µg/m^3^ (Guan et al. 2016)). In contrast, few studies have been conducted on short-term O_3_ exposure; no studies have analyzed the relationship between short-term O_3_ exposure and AMI hospitalization in China. Therefore, we conducted a multicenter time-series study in Henan Province, China, to explore the effect of short-term O_3_ exposure on AMI hospitalization. In addition, this study quantified the fraction of hospitalizations attributable to O_3_; the number of AMI-related hospitalizations and hospitalization costs saved by falling below China’s national secondary air quality standards was quantified to make appropriate recommendations for relevant public health policies.

## Materials and methods

### Study population

In this study, data were obtained from seven large general hospitals (Table S1) in Henan Province, China (31°23′–36°22′N, 110°21′–116°39′E), located in five cities: Anyang, Xinxiang, Luoyang, Kaifeng, and Nanyang (Figure S1). The coordinates of the monitoring stations and hospitals were determined on regional maps using ArcGIS (version 10.5). The selected hospitals were ranked as the top tertiary hospitals in prefecture-level cities and were located in densely populated areas with convenient transportation. With advanced equipment, complete medical departments, and strong diagnosis and treatment capabilities, they have a reputation for diagnosing and treating cardiovascular diseases. Therefore, they reflect the incidence of common cardiovascular diseases in the local area (Wang et al. 2020a). Between January 1, 2016, and October 31, 2021, 24,489 patients with AMI in the study sites mentioned above were enrolled in this study; all acute myocardial infarctions were clinically diagnosed and validated (Thygesen et al. 2018) with diagnosis code I21, from the 10th edition of the International Classification of Diseases (ICD-10). We collected information on these inpatients, including hospitalization number, inpatient medical records, sex, age, home address, and first diagnosis information. Each patient’s hospital address was geocoded to longitude and latitude and matched to the nearest monitoring station (Table S2). These data were also categorized according to sex, age, season, and sunshine duration. The age groups were as follows: younger (15–64 years) and older adults (≥ 65 years). The cold season was from November to April (next year), and the warm season was from May to October. We set the short (≤ 6.3 h) and long sunshine duration groups (> 6.3 h) based on the average sunshine duration in the warm season (6.33 h).

### Air pollution and weather data

Air pollutant concentrations in the study area: O_3_, particulate matter with aerodynamic matter ≤ 2.5 μm (PM_2.5_), particulate matter with aerodynamic matter ≤ 10 μm (PM_10_), carbon monoxide (CO), nitrogen dioxide (NO_2_), and sulfur dioxide (SO_2_) from the China General Environmental Monitoring Station (http://www.cnemc.cn/). Air pollution data were collected from the 28 fixed-site monitoring stations mentioned above, located away from emission sources, such as roads, industries, and factories, and whose measurements reflect overall city averages rather than local pollution sources. For PM_2.5_, PM_10_, CO, NO_2_, and SO_2_, the daily concentrations are 24-h averages, while the diurnal concentrations of O_3_ vary significantly; therefore, the maximum 8-h averages of all effective monitoring stations were selected (Liu et al. 2022).

Meteorological data were obtained from the China National Meteorological Information Center (http://data.cma.cn/). Two meteorological parameters (daily average relative humidity and daily average temperature) were introduced as covariates in the model to adjust for confounding factors. This study was approved by the ethical review committee of The First Affiliated Hospital of Xinxiang Medical University (No. 2020097; Xinxiang, China).

### Statistical analysis

#### Modeling the association between O_3_ and AMI hospitalizations and subgroup analysis

Daily AMI hospitalizations, O_3_, and meteorological data were linked by admission date for time-series analysis. The number of admissions of patients with AMI followed a Poisson distribution. A proposed Poisson regression model based on a generalized summation linear model and a distributed lag nonlinear model (DLMN) was used to evaluate the association between air pollutants and AMI hospitalizations (Chang et al. 2022). The association between AMI hospitalizations and O_3_ was fitted using the “DLMN” analysis package in R software (version 4.12). This was combined with the Akaike information criterion (Dziak et al. 2020). We included the following covariates in the model: a natural cubic spline (NS) function with 2 degrees of freedom (df) per year was used to control for long-term trends and seasonality; the effects of humidity and temperature were controlled with 2 and 5 df NS functions, respectively; the day-of-week effect (Dow) was set as a covariate; and the Chinese holiday effect was also considered in the model to eliminate the effect of public holidays.

The algebraic expression of the model is as follows:

$${\text{Log}}\left(E\left({Y}_{t}\right)\right)=\alpha +\beta {X}_{t,l}+ns\left(\mathrm{times},2\right)+ns\left(\mathrm{hum},2\right)+ns\left(\mathrm{temp},5\right)+ \mathrm{Dow}+ \mathrm{holidays}$$

where $E\left({Y}_{t}\right)$ is the number of AMI hospitalizations on day *t*, $\alpha$ is the intercept, and $\beta {X}_{t,l}$ is the cross-basis matrix for air pollution. *ns*() represents the NS function.

To explore the lag association between AMI hospitalizations and O_3_, we set the pollutant as a cross-matrix and used a polynomial function for the lags. We set the lag days to 7 days (Raza et al. 2018; Stafoggia et al. 2010) and rated the lagged cumulative effect of each 10 µg/m^3^ increase in airborne O_3_ concentration using single-day lag (lag0–lag7) and multi-day cumulative lags (lag01–lag07). In addition, we stratified the study by sex (male and female), age (young and older patients), season (warm and cool seasons), and warm season sunshine duration (long and short sunshine durations) to identify potentially susceptible subgroups for O_3_ exposure.

#### Calculation of AMI hospitalizations, hospitalization days, and hospitalization costs attributable to O_3_

Based on the attribution fraction calculation method in previous studies (Chen et al. 2017a; Cheng et al. 2017; Deubner et al. 1975), we estimated the total attribution fraction (AF) and the number of attributions (AN) for AMI hospitalizations. Therefore, the formulae are as follows:

1 $$AF=1 - \mathrm{exp}(-\beta )$$

2 $$A{N}_{i} = A{F}_{i}{*N}_{i}$$

where $AF$ is the attribution fraction for O_3_, $A{N}_{i}$ is the number of attributions on day *i*, and ${N}_{i}$ is the total number of AMI hospitalizations, hospitalization days, and hospitalization costs. According to Steenland and Armstrong (2006), formulae (1) can be simplified to AF = (RR—1)/RR, RR is the cumulative maximum relative risk.

#### Assessment of the potential health and economic effects of O_3_ concentrations below the current Chinese ambient air quality standard for O_3_ (8 h: 160 µg/m^3^) during the study period

Daily O_3_ concentrations below the secondary standard for ambient air quality in China (8 h: 160 µg/m^3^) accounted for 84.2% (1795/2131) of the total study period. We maintained the O_3_ concentrations in the range of 10‒160 µg/m^3^ (every 30 unit increment). The potential health and economic effects were estimated based on five O_3_ concentrations (10–40–70–100–130 µg/m^3^). The magnitude of the health benefit (i.e., the number of preventable cases per year) was measured as the difference between the annual number of attributed cases at the O_3_ concentration level of the Chinese ambient air quality secondary standard and the number of cases below the five O_3_ concentration limits developed. Similarly, the magnitude of the economic benefit, that is, the annual hospitalization cost savings (in RMB), is measured as the difference between the annual attributable hospitalization cost at the O_3_ concentration level of the Chinese ambient air quality secondary standard and the hospitalization cost below the five established O_3_ concentration limits.

We used a cumulative lag of 3 days for risk assessment in constructing the dual-pollution model, subgroup analysis, and assessment of population attribution because O_3_ produced the most significant effect estimate at a cumulative lag of 3 days.

#### Sensitivity analysis

First, we constructed a dual-pollution model to test the effect of using the lag of the co-pollution model by adjusting for common pollutants (PM_2.5_, PM_10_, CO, NO_2_, and SO_2_) in the model. Second, the stability of the results was tested by varying the df values (3, 4, 5, 6, and 7) of the long-term trends.

The statistical analysis for this study was performed using the “dlnm” package in R software (version 4.12). This function reports the effect of every 10 µg/m^3^ increase in O_3_ on the daily AMI hospitalizations, estimated as relative risk (RR) and 95% confidence interval (95% CI). A two-sided test *p*-value < 0.05 was considered statistically significant.

## Results

### Study patients and exposure characteristics

Table 1 shows the descriptive statistics for daily AMI hospitalizations, hospitalization days, total hospitalization costs, meteorological factors, and air pollution during the study period. Between January 1, 2016, and October 31, 2021, we recorded 24,489 patients diagnosed with AMI (ICD10: I21) in these hospitals, with an average of 11.49 admissions daily. The patients were predominantly male (8.35 cases daily) and aged 15–64 years (6.18 cases daily). The daily maximum 8-h average O_3_ concentration during the study period was 104.29 µg/m^3^ (range:4–277 µg/m^3^).

**Table 1:** Descriptive statistics of daily AMI hospitalizations, air pollution, and meteorological variables in Henan, China, during 2016–2021

	Mean ± SD	Min	25^th^	50^th^	75^th^	Max
Hospitalizations (counts)						
Total	11.49 ± 4.61	1.00	8.00	11.00	14.00	36.00
Males	8.35 ± 3.67	0.00	6.00	8.00	10.00	30.00
Females	3.14 ± 2.02	0.00	2.00	3.00	4.00	14.00
Young	6.18 ± 2.89	0.00	4.00	6.00	8.00	24.00
Elderly	5.31 ± 2.90	0.00	3.00	5.00	7.00	18.00
Hospitalizations (stays in days)						
Total	134.79 ± 55.90	4.00	96.00	128.00	166.00	441.00
Males	97.42 ± 44.82	0.00	65.00	92.00	124.00	375.00
Females	37.37 ± 27.61	0.00	17.00	32.00	52.00	233.00
Young	72.10 ± 36.03	0.00	46.00	67.00	94.00	262.00
Elderly	62.67 ± 37.34	0.00	36.00	57.00	83.00	262.00
Hospitalizations (costs in 1000 RMB)						
Total	357.84 ± 163.79	8.15	243.85	334.53	450.26	1234.26
Males	268.2 ± 135.46	0.00	170.28	251.86	343.79	888.85
Females	89.65 ± 73.46	0.00	37.50	74.52	127.87	943.94
Young	205.46 ± 113.24	0.00	123.97	189.26	271.56	822.75
Elderly	152.32 ± 101.53	0.00	77.46	136.30	206.27	988.01
Meteorological factors						
Average temperature (℃)	16.06 ± 9.78	-10.20	7.60	16.90	25.00	34.60
Relative humidity (%)	61.61 ± 19.20	9.00	47.00	63.00	77.00	100.00
Sunshine duration (h)	5.46 ± 3.93	0.00	1.40	6.00	8.80	13.50
Air pollutants						
O_3_ (µg/m^3^)	104.29 ± 52.44	4.00	62.00	99.00	142.00	277.00
PM_2.5_ (µg/m^3^)	62.01 ± 50.89	3.00	30.00	45.00	76.00	665.00
PM_10_ (µg/m^3^)	110.05 ± 72.06	6.00	63.00	92.00	135.00	915.00
CO (mg/m^3^)	1.18 ± 0.69	0.20	0.80	1.00	1.40	10.20
NO_2_ (µg/m^3^)	36.53 ± 18.27	5.00	22.00	33.00	48.00	168.00
SO_2_ (µg/m^3^)	17.97 ± 16.6	2.00	8.00	13.00	22.00	176.00

*AMI* acute myocardial infarction; young: 15–64 years; elderly: 65 + years; *O*_*3*_ ozone; *PM*_*2.5*_ particulate matter with aerodynamic matter ≤ 2.5 μm; *PM*_*10*_ particulate matter with aerodynamic matter ≤ 10 μm; *CO* carbon monoxide; *NO*_*2*_ nitrogen dioxide; *SO*_*2*_ sulfur dioxide; *RMB* Chinese dollar, 1000 RMB ≈ 147 USD in January 2023.

**Fig. 1:**
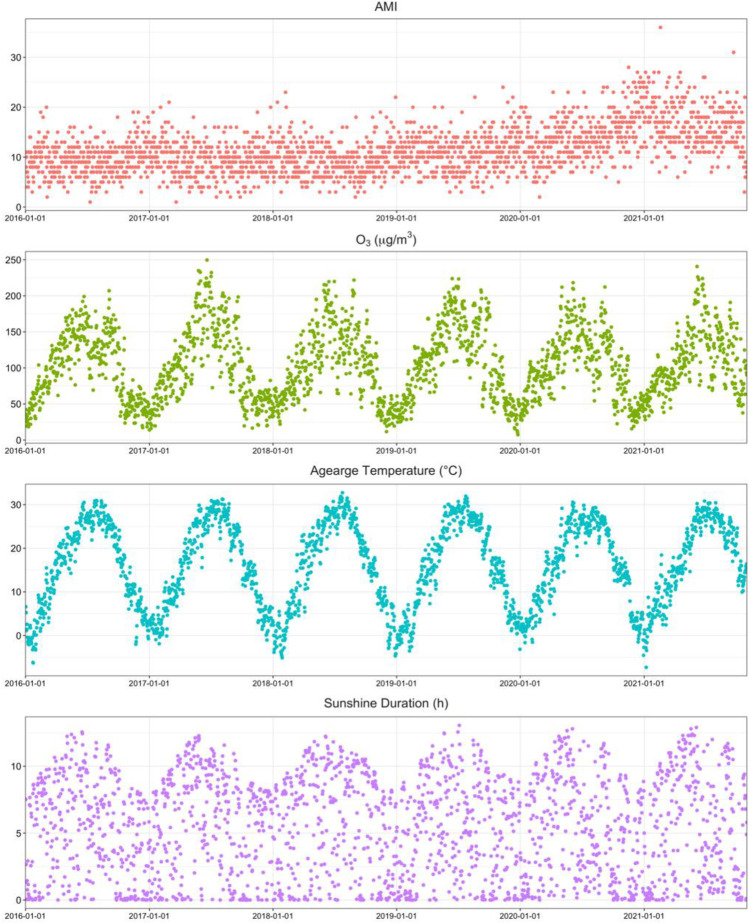
The distribution of O_3_, meteorological variables, and AMI hospitalizations in Henan, China, during 2016–2021. AMI, acute myocardial infarction; O_3_, ozone

Figure 1 shows the time series patterns of daily AMI hospitalizations, O_3_ concentrations, and meteorological indicators between 2016 and 2021. The number of AMI admissions showed seasonal characteristics of high in winter and low in summer, with an overall increasing trend between 2016 and 2021. O_3_, average daily temperature, and sunshine duration showed clear seasonal patterns of variation. Figure S2 shows the interval percentages of O_3_ concentrations and monthly O_3_ trends in the different cities during the study period. Table S3 shows the Spearman correlations between all pollutant indicators and some meteorological indicators, indicating a significant positive correlation between O_3_ and temperature (*r* = 0.78), the same positive correlation with sunshine duration (*r* = 0.54), and a negative correlation between O_3_ and the remaining air pollution indicators.

### Effects of O_3_ in single- and dual-pollutant models

Table S4 shows the association between different air pollutants of O_3_, PM_2.5_, PM_10_, CO, NO_2_, and SO_2_ and AMI hospitalization at different lag days, with short-term O_3_ exposure being the most significantly associated with increased risk of AMI hospitalization in this study area compared to other air pollutants. Table 2 and Figure S3 show the single- and dual-pollution models of the association between O_3_ on different lag days and AMI hospitalization. We observed that lag0, lag1, and lag (01–07) were all risk factors for AMI hospitalization in the single- and dual-pollution models, with statistically significant differences and the largest effect at lag03 (RR = 1.132, 95% CI:1.083–1.182).

**Table 2:** RR (95% CI) of AMI hospitalizations expose to O_3_ single- and dual-pollution model (every 10 µg/m^3^ increase) at different lag days

	O_3_		O_3_ + PM_2.5_		O_3_ + PM_10_		O_3_ + CO		O_3_ + NO_2_		O_3_ + SO_2_	
	RR	95% CI	RR	95% CI	RR	95% CI	RR	95% CI	RR	95% CI	RR	95% CI
Lag0	**1.076**	**(1.043–1.111)**	**1.074**	**(1.035–1.114)**	**1.069**	**(1.031–1.109)**	**1.077**	**(1.038–1.117)**	**1.062**	**(1.024–1.102)**	**1.060**	**(1.022–1.099)**
Lag1	**1.037**	**(1.022–1.053)**	**1.033**	**(1.015–1.051)**	**1.031**	**(1.013–1.049)**	**1.032**	**(1.014–1.05)**	**1.030**	**(1.012–1.048)**	**1.034**	(1.016–1.051)
Lag2	1.013	(0.997–1.029)	1.008	(0.989–1.027)	1.008	(0.989–1.027)	1.006	(0.987–1.025)	1.010	(0.991–1.029)	1.015	(0.996–1.034)
Lag3	1.000	(0.988–1.013)	0.995	(0.980–1.010)	0.996	(0.981–1.011)	0.993	(0.979–1.008)	0.999	(0.984–1.014)	1.003	(0.988–1.018)
Lag4	0.995	(0.983–1.008)	0.99	(0.975–1.004)	0.991	(0.977–1.006)	0.989	(0.974–1.003)	0.993	(0.979–1.008)	0.995	(0.981–1.01)
Lag5	0.995	(0.980–1.011)	0.989	(0.971–1.007)	0.990	(0.972–1.009)	0.988	(0.97–1.006)	0.991	(0.973–1.009)	0.991	(0.973–1.009)
Lag6	0.997	(0.985–1.01)	0.988	(0.973–1.003)	0.990	(0.975–1.005)	0.986	(0.971–1.001)	0.988	(0.973–1.003)	0.989	(0.974–1.004)
Lag7	0.999	(0.973–1.025)	0.984	(0.955–1.015)	0.986	(0.957–1.017)	0.978	(0.949–1.009)	0.983	(0.953–1.013)	0.987	(0.958–1.018)
Lag01	**1.116**	**(1.073–1.161)**	**1.109**	**(1.060–1.160)**	**1.102**	**(1.054–1.153)**	**1.111**	**(1.062–1.162)**	**1.094**	**(1.046–1.144)**	**1.095**	**(1.047–1.146)**
Lag02	**1.131**	**(1.085–1.179)**	**1.118**	**(1.066–1.173)**	**1.111**	**(1.059–1.165)**	**1.117**	**(1.065–1.172)**	**1.104**	**(1.053–1.158)**	**1.112**	**(1.060–1.166)**
Lag03	**1.132**	**(1.083–1.182)**	**1.112**	**(1.058–1.169)**	**1.107**	**(1.053–1.163)**	**1.110**	**(1.056–1.167)**	**1.103**	**(1.049–1.160)**	**1.115**	**(1.061–1.172)**
Lag04	**1.126**	**(1.077–1.178)**	**1.101**	**(1.046–1.158)**	**1.097**	**(1.042–1.154)**	**1.097**	**(1.043–1.154)**	**1.096**	**(1.041–1.153)**	**1.110**	**(1.054–1.168)**
Lag05	**1.121**	**(1.070–1.175)**	**1.088**	**(1.031–1.148)**	**1.086**	**(1.029–1.146)**	**1.084**	**(1.027–1.143)**	**1.086**	**(1.029–1.146)**	**1.099**	**(1.042–1.161)**
Lag06	**1.118**	**(1.064–1.176)**	**1.075**	**(1.016–1.137)**	**1.075**	**(1.016–1.138)**	**1.068**	**(1.009–1.130)**	**1.073**	**(1.014–1.136)**	**1.087**	**(1.027–1.151)**
Lag07	**1.117**	**(1.064–1.172)**	**1.058**	**(1.002–1.117)**	**1.061**	**(1.004–1.120)**	**1.045**	**(0.989–1.103)**	**1.054**	**(0.998–1.114)**	**1.073**	**(1.016–1.134)**

Bold: *p* < 0.05.

**Fig. 2:**
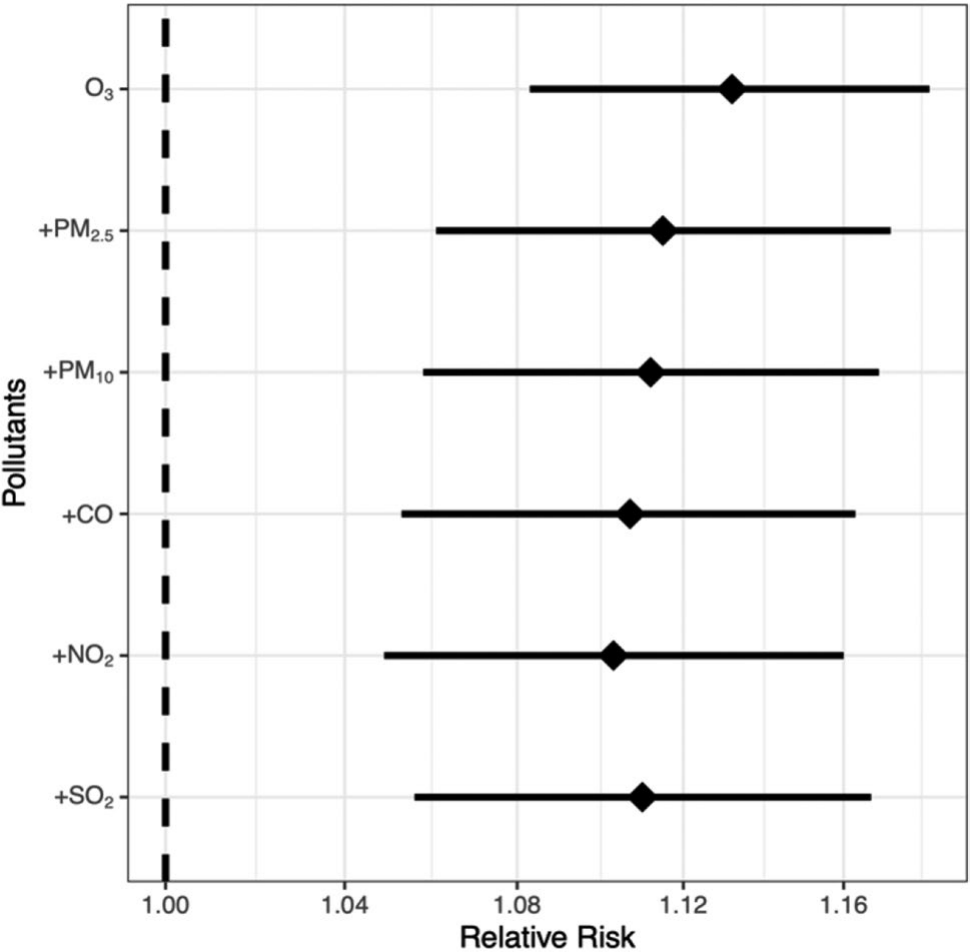
RR (95% CI) of AMI hospitalizations for every 10 µg/m.^3^ increase in O_3_ in the dual-pollutant model at lag 03

Figure 2 shows the results of the dual-pollution model at lag03, which are insignificantly different from those of the O_3_ single-pollution model.

### Associations between O_3_ and AMI in different subgroups

**Fig. 3:**
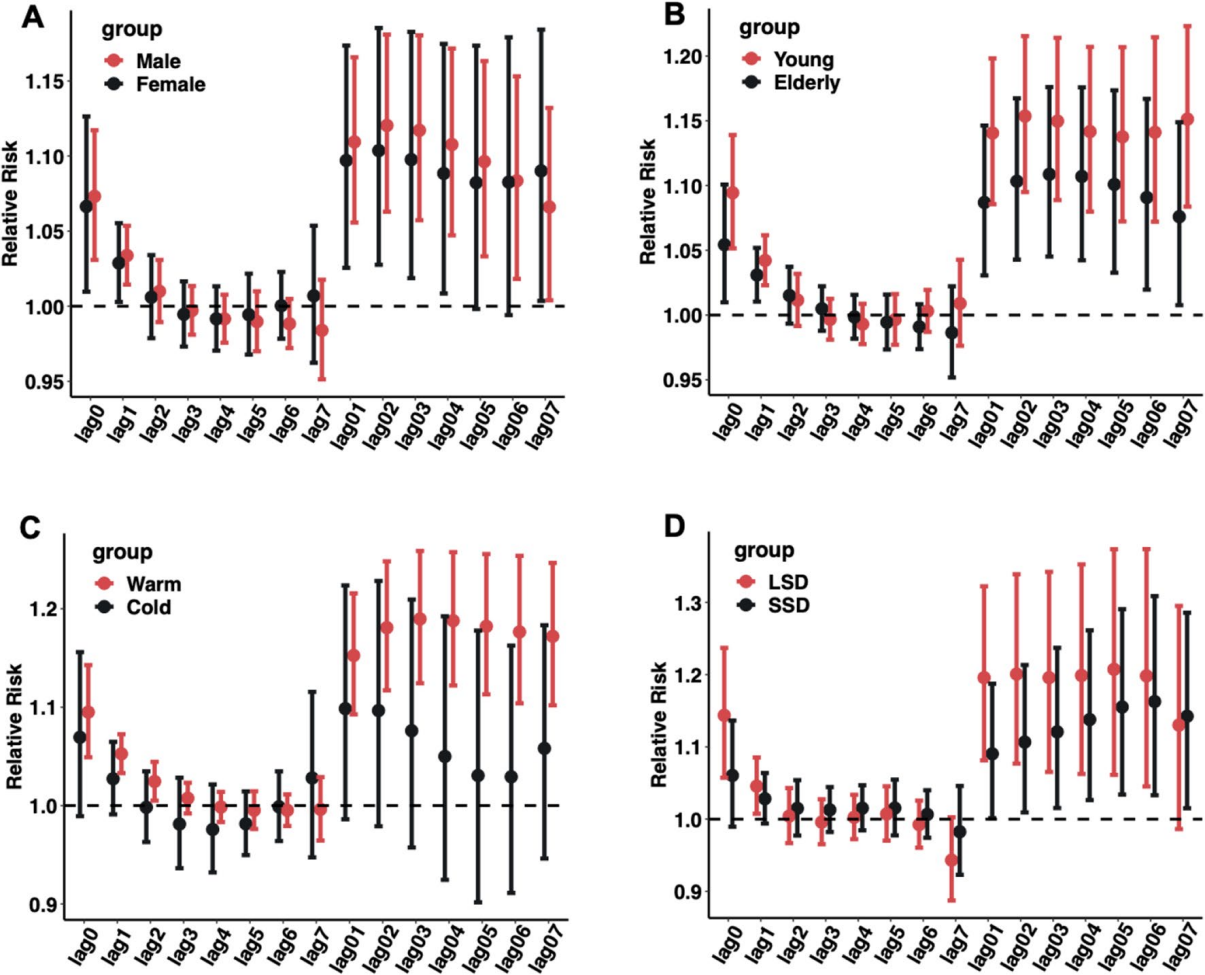
RR (95% CI) of AMI hospitalizations expose to O_3_ (every 10 µg/m^3^ increase) in different subgroups at different lag days. **A** The single-day and cumulative relative risk effects in males and females. **B** The single-day and cumulative relative risk effects in young and elderly. **C** The single-day and cumulative relative risk effects in warm season and cold season. **D** The single-day and cumulative relative risk effects of LSD (long sunshine duration) and SSD (short sunshine duration) in warm season

Figure 3 reports the RR (95% CI) values of O_3_-related AMI hospitalizations by sex, age, season, and different sunshine hours in the warm season. The mean sunshine duration in the warm season is shown in Table S6 as 6.33 h; we defined warm season sunshine duration ≤ 6.3 has short sunshine duration (SSD) and > 6.3 has long sunshine duration (LSD) (Table S7). Among the different subgroups, AMI hospitalizations in the male, younger patients, warm season, and LSD groups were more susceptible to O_3_ effects.

In the sex stratification, the largest effect of O_3_ on AMI hospitalizations was estimated in lag03 (RR = 1.117, 95% CI:1.058–1.180) for males and lag02 (RR = 1.104, 95% CI:1.028–1.185) for females. In age stratification, the largest effect of O_3_ on AMI hospitalizations was estimated in lag02 (RR = 1.154, 95% CI:1.095–1.215) for younger patients and lag03 (RR = 1.109, 95% CI:1.054–1.176) for older adult patients. In seasonal stratification, the largest effect value of O_3_ on AMI hospitalizations during the warm season was estimated at lag03 (RR = 1.190, 95% CI:1.124–1.259); the difference was not statistically significant in the cold season. In the stratification of different daylight hours in the warm season, the largest effect value of O_3_ on AMI hospitalizations in the LSD group was estimated at lag02 (RR = 1.201, 95% CI:1.077–1.339) and in the SSD group at lag06 (RR = 1.163, 95% CI:1.015–1.286) (Table S5).

**Fig. 4:**
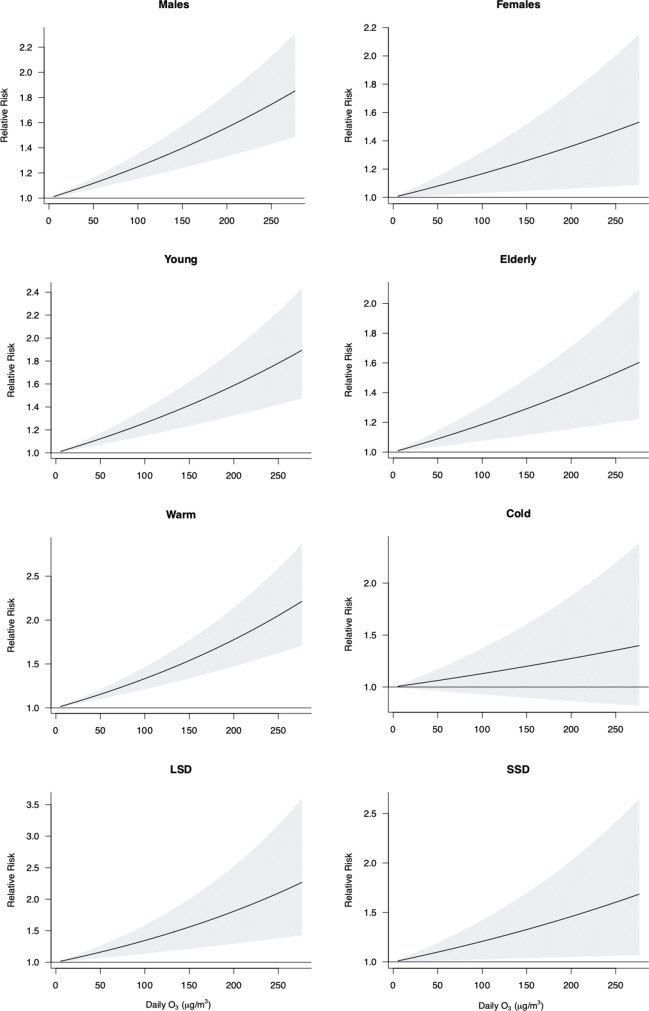
Exposure–response curves in different subgroups of O_3_ with AMI hospitalizations at lag 03. LSD, long sunshine duration (sunshine duration > 6 h); SSD, short sunshine duration (sunshine duration ≤ 6 h); both LSD and SSD are warm season sunshine durations

Figure 4 shows the exposure–response curves between O_3_ and AMI hospitalizations in different subgroups at lag03. Except for the curves in the cold season, which were not statistically significant, the O_3_ curves showed a linear trend in all subgroups, and the cumulative RR increased with increasing O_3_ concentration.

### Attributable burden of AMI hospitalizations due to O_3_

Table 3 shows the total attributable fraction (AF) and attributable number (AN) of AMI and different subgroups of hospitalizations attributable to O_3_. We estimated that O_3_ accounted for 11.66% of the total cases (2,856), 33,492 days of total hospitalization, and 90 million RMB of the total hospitalization costs. Among the different subgroups, the AF and AN were higher in males, younger patients, and warm seasons. When we adjusted for the dual air pollution factors, the AF decreased slightly, as shown in Table S8.

**Table 3:** Fraction and number of AMI hospitalizations, hospital stays, and hospitalization costs attributable to O_3_

	Attributable fraction of hospitalizations (%)		Attributable number of hospitalizations (no.)		Attributable number of hospital stays (days)		Attributable number of hospitalization costs (RMB, million)	
Total	11.66	(7.66–15.40)	2856	(1878–3768)	33,492	(22,014–44,226)	90	(60–120)
Males	10.47	(5.39–15.25)	702	(360–1020)	8,340	(4296–12,144)	18	(12–30)
Females	8.93	(1.86–15.47)	1590	(330–2754)	18,528	(3879–32,112)	54	(12–90)
Young	13.04	(8.17–17.63)	1476	(924–1992)	17,418	(10,914–23,538)	42	(24–60)
Elderly	9.83	(4.31–14.97)	1296	(570–1968)	15,102	(6618–22,992)	42	(18–66)
Warm	15.97	(11.03–20.57)	1620	(1122–2088)	18,978	(13,110–24,450)	48	(36–66)

Figure S4 illustrates that if O_3_ concentrations could be reduced to levels below current Chinese ambient air quality standards during the study, such as maintaining 130 µg/m^3^, 185 AMI cases could be prevented yearly, resulting in savings of 6 million RMB. If O_3_ were maintained at even lower levels (below 10 µg/m^3^), 1056 AMI hospitalizations could be prevented yearly, saving more than 30 million RMB and yielding substantial health and economic benefits. These results varied significantly across the subgroups.

### Sensitivity analyses

The sensitivity analysis results are reported in Table S9; we varied the df range of the long-term trend (3, 4, 5, 6, 7) between total AMI hospitalizations, sex, age, and different seasons and found similar results to our main analysis, indicating that the outcome estimates are robust.

## Discussion

This study was performed in the north-central provinces of China, with four seasons of the year. It is representative of China as a major agricultural province with low per capita income levels, and is one of the most polluted provinces in China (Zhang et al. 2022). We first constructed single-pollution models for different air pollutants of O_3_, PM_2.5_, PM_10_, CO, NO_2_, and SO_2_ and the risk of AMI hospitalization (Table S4), and found that short-term O_3_ exposure was associated with a more significant increase in the risk of AMI hospitalization than other air pollutants, and therefore, the analysis was focused on short-term O_3_ pollution exposure. We found that AMI hospitalization and short-term O_3_ exposure in Henan were significantly associated with a lag effect, with the greatest risk at a cumulative lag of 3 days (RR = 1.132, 95% CI:1.083–1.182) and a linear exposure–response curve (Figure S3). In addition, males, younger patients, warm season, and LSD groups were more susceptible to O_3_ levels. To our knowledge, this is the first time that DLMN analysis has been used to explore the relationship between short-term O_3_ exposure and AMI hospitalization in China and to attempt to quantify the number of O_3_-attributable AMI hospitalizations, hospitalization days, and hospitalization costs.

In epidemiological studies, O_3_ exposure is associated with the risk of cardiovascular and cerebrovascular diseases and mortality (Nuvolone et al. 2013; Raza et al. 2019; Wen et al. 2022; Zhang et al. 2019, 2021). A multicenter study from Italy showed that short-term exposure to O_3_ increased all-cause mortality in a population with an RR of 1.0043 (95% CI:1.0029, 1.0057) (Wen et al. 2022). Raza et al. (2019) found that short-term exposure to O_3_ increases the risk of out-of-hospital cardiac arrest. Henrotin et al. (2010) showed that short-term exposure to even low levels of O_3_ may cause ischemic cerebrovascular events and myocardial infarction (MI) and was most significant at a cumulative lag of 3 days with an RR of 1.115 (95% CI:1.027–1.209), consistent with our findings; however, Henrotin et al. included fewer patients with MI. Furthermore, it has been shown that there is no significant association between hourly O_3_ exposure and the onset of acute coronary syndromes (Chen et al. 2022), which may be due to various reasons, such as the study period, study site, and differences in O_3_ exposure levels. Most previous studies on O_3_ and cardiovascular disease were conducted in developed countries, where the mean 8-h O_3_ concentrations were generally below 60 µg/m^3^, which is significantly lower than the mean 8-h O_3_ concentration in our study area (104.3 µg/m^3^). The outcome supports this study’s results and points to a need for further control of O_3_ levels to lower levels in our study area.

Because pollutants in the atmosphere usually exist simultaneously, we constructed a dual-pollution model for PM_2.5_, PM_10_, CO, NO_2,_ and SO_2_, and included covariates such as temperature, humidity, Dow, and public holidays in the model. The results showed no significant difference compared with the single-pollution model for O_3_, and the RR of the dual-pollution model was slightly lower than that of the single-pollution model, probably due to the significant negative correlation between O_3_ and other air pollutants (Table S3). O_3_ and temperature were significantly positively correlated (*r* = 0.78), with significantly higher O_3_ concentrations in the warm season than in the cold season (Table S6). The results of the subgroup analysis indicated that short-term exposure to O_3_ was associated with a significantly higher risk of AMI hospitalization in the warm season than in the cold season, similar to the findings of Wen et al. (2022). Similarly, we found that O_3_ and sunshine duration were also positively correlated (*r* = 0.54), which may be related to the involvement of ultraviolet light in the formation of O_3_ (Chang et al. 2022; Wang et al. 2020b). As we found that the association between O_3_ and the risk of AMI hospitalization was insignificant in the cold season, we further divided the patients into the LSD and SSD groups according to the mean sunshine hours in the warm season (6.33 h) (Table S7). We found that patients in the LSD group were associated with a higher risk of AMI, supporting the association between O_3_ concentration and the risk of AMI hospitalization. Concerning the age and sex of the susceptible population, the findings of current studies are mostly inconsistent; Wen et al. in Italy did not find differences between age and sex groups exposed to O_3_ and all-cause mortality (Wen et al. 2022). In a multi-community study, Schwartz et al. reported that for every 10 µg/m^3^ increase in O_3_ (mean 0–2 day lag), cardiovascular disease mortality was higher in people aged ≥ 65 years than in younger people, and females and blacks were more likely to be affected (Medina-Ramón and Schwartz 2008). In this study, we have found that males and younger patients are more likely to be affected by O_3_, possibly because there were more males and patients with AMI aged 14–64 years in this study and that this population would be more likely to expose themselves to higher levels of O_3_ through outdoor activities or by engaging in occupations (such as policeman and delivery-man) (Chang et al. 2022); at the meantime, males were more likely to suffer from respiratory disease (Wang et al. 2022b), which in turn affects cardiorespiratory function, which may also increase the risk of AMI hospitalization due to O_3_ exposure in this population.

The mechanism of O_3_ and the occurrence of AMI is unclear, and the results of some studies are inconsistent (Goodman et al. 2015). Michaudel et al. found that O_3_ dissolved in plasma generates reactive oxygen species (Michaudel et al. 2018), causing apoptosis of endothelial cells and damaging blood vessels (Emon et al. 2021). In addition, a study on healthy individuals showed that exposure to O_3_ resulted in elevated plasma levels of inflammatory markers (Kim et al. 2011; Pirozzi et al. 2015) and that inflammatory mediators can enter the circulatory system and stimulate platelet activation (Day et al. 2017) and the release of coagulation factors (Münzel et al. 2017), increasing the chances of thrombosis and promoting coronary events. O_3_ exposure may also be associated with changes in the autonomic nervous system (Arjomandi et al. 2015; Jia et al. 2011) and blood pressure (Niu et al. 2022); however, some studies have found a protective effect of O_3_ on ischemic myocardium (Wang et al. 2022a; Yu et al. 2021).

To complement the traditional exposure risk assessment, we evaluated the number of AMI hospitalizations, hospitalization days, and hospitalization costs attributable to O_3_. The results showed that 11.66% of AMI hospitalizations were attributable to the effects of O_3_ in the environment, equivalent to 2856 patients and 90 million RMB (Table 3). A modelling study of 12,946 cities included globally showed that the fraction of global O_3_-attributable mortality occurring in urban areas gained in their share of global O_3_-attributable burden from 2000 to 2019 (from 35 to 37%) (Malashock et al. 2022). Another study of the global burden of chronic obstructive pulmonary disease (COPD) attributable to ambient O_3_ exposure, conducted in 204 countries around the world, showed that among COPD-related deaths, attributable O_3_ exposure is generally on the rise globally, from 8.22% in 1990 to 11.13% in 2019 (Wang et al. 2022b). In contrast, a study by Chen et al. (2017b) in area of Zhejiang Province, China, found that the population attributable fractions for O_3_ exposure to COPD is 5.32%. This study fills a research gap in China in terms of AMI hospitalizations attributable to O_3_ exposure. The medical burden on local hospitals is enormous, and the local economic loss to the government and patients is huge. The Chinese government promulgated the Ambient Air Quality Standard (GB 3095–2012) in 2012, in which the secondary standard applicable to general areas sets the 8-h average concentration of O_3_ at 160 µg/m^3^; even below this standard, short-term exposure to O_3_ exhibits a significant association with an increased risk of AMI hospitalization. In September 2021, the World Health Organization released the latest unprecedentedly stringent “Air Quality Guidelines” (AQG) (Burki 2021), which set higher requirements for atmospheric O_3_ concentrations and added a new indicator of O_3_ concentrations less than 60 µg/m^3^ in the warm season, compared to the warm season in Henan, where O_3_ concentrations are much higher than this standard. If more stringent measures could be implemented to control O_3_ to lower concentrations, as shown in Figure S4, hundreds of AMI cases could be prevented yearly, and millions of RMB could be saved. This study is the first to analyze the relationship between short-term exposure to O_3_ and the risk of AMI hospitalization in areas of China with more severe O_3_ pollution, providing a basis for the prevention of AMI and environmental improvement in Henan Province.

Our study had some limitations. First, as an environmental ecological study, we could not include more comprehensive information on individuals in our study, such as disease history, complications, biochemical indicators, and medication use, which will need to be included in future in-depth clinical studies. Second, owing to the nature of the time-series study design and the uneven distribution of air monitoring stations in some study areas, there was variability in O_3_ exposure per patient, which may have led to reduced variability in exposure conditions and biased results. Third, in practical studies, the AF calculations we performed were often not univariate, and also, observational studies introduce certain confounding effects. This leads to some deviation from the true AF when we calculate AF. Fourth, we did not consider information related to hospitalization reimbursement for each patient with AMI when calculating the annual hospitalization costs that could be reduced by reducing O_3_ and the fact that reimbursement may vary across regions, which may have affected our estimates of O_3_-induced costs. Fifth, this study was conducted in only one province in central China; therefore, caution should be taken when generalizing to populations in other regions or countries.

## Conclusions

We found that short-term exposure to O_3_ was significantly associated with an increased risk of hospitalization for AMI and that the exposure–response curve was linear at a cumulative lag of 3 days, particularly for males, younger patients, warm season, and long sunshine duration with greater risk. Further reductions in O_3_ levels, particularly in developing countries, may bring substantial health and economic benefits to patients and local healthcare institutions.

## Supplementary Information

Below is the link to the electronic supplementary material.

Supplementary file1 (DOCX 872 KB)

## Data Availability

All data were presented in our analytical results or please contact the first author or the corresponding author on reasonable request.
