# Supplementary material for: A time-series analysis of short-term ambient ozone exposure and hospitalizations from acute myocardial infarction in Henan, China
Source: Environ Sci Pollut Res Int. 2023 Jul 28;30(40):93242–54. doi: 10.1007/s11356-023-28456-2 (PMC10447277; doi:10.1007/s11356-023-28456-2)
Supplement: Supplementary file 1 — Supplementary file1 (DOCX 872 KB) [file 11356_2023_28456_MOESM1_ESM.docx]

**Supplementary Appendix**

**Contents**

Supplementary Figure 1. The locations of air pollutants monitoring stations and hospitals in Henan, China 02

Supplementary Figure 2. Association between O_3_ single- and dual-pollution model (every 10 µg/m^3^ increase) and AMI hospitalization at single-day lag 03

Supplementary Figure 3. Percentage of O_3_ concentration interval in Henan Province during the study period (A) and average O_3_ concentration per month from 2016-2021 in different cities (B) 04

Supplementary Figure 4. The estimated annual preventable AMI hospitalizations (A) and annual savable hospitalization costs (B) if the historical O_3_ concentration could be reduced to a relatively low level 05

Supplementary Table 1. Coordinates of each hospital in the study area 06

Supplementary Table 2. Coordinates of each city air monitoring station in the study area 07

Supplementary Table 3. Spearman’s correlation coefficients between daily air pollutants (O_3_: 8h) and meteorological data in Henan, 2016–2021 08

Supplementary Table 4. RR (95% CI) of AMI hospitalizations with an increase of 10μg/m^3^ in air pollutants (and 1mg/m^3^ in CO) according to the single-pollutant model at different lag days. 09

Supplementary Table 5. RR (95% CI) of AMI hospitalizations at different lag days with O_3_ (every 10 µg/m^3^ increase) in different subgroups 10

Supplementary Table 6. Comparison of the AMI hospitalizations, O_3_ concentration and mean temperature in cold and warm seasons 11

Supplementary Table 7. Comparison of the AMI hospitalizations, O_3_ concentration, average temperature and sunshine duration in warm seasons 12

Supplementary Table 8. Attributable fraction of hospitalizations to single- and dual-pollution models of O_3_ exposure (%) 13

Supplementary Table 9. Results of sensitivity analyses by changing degree of freedom for long-term trend and seasonality 14

**Supplementary Figure 1. The locations of air pollutants monitoring stations and hospitals in Henan, China.**


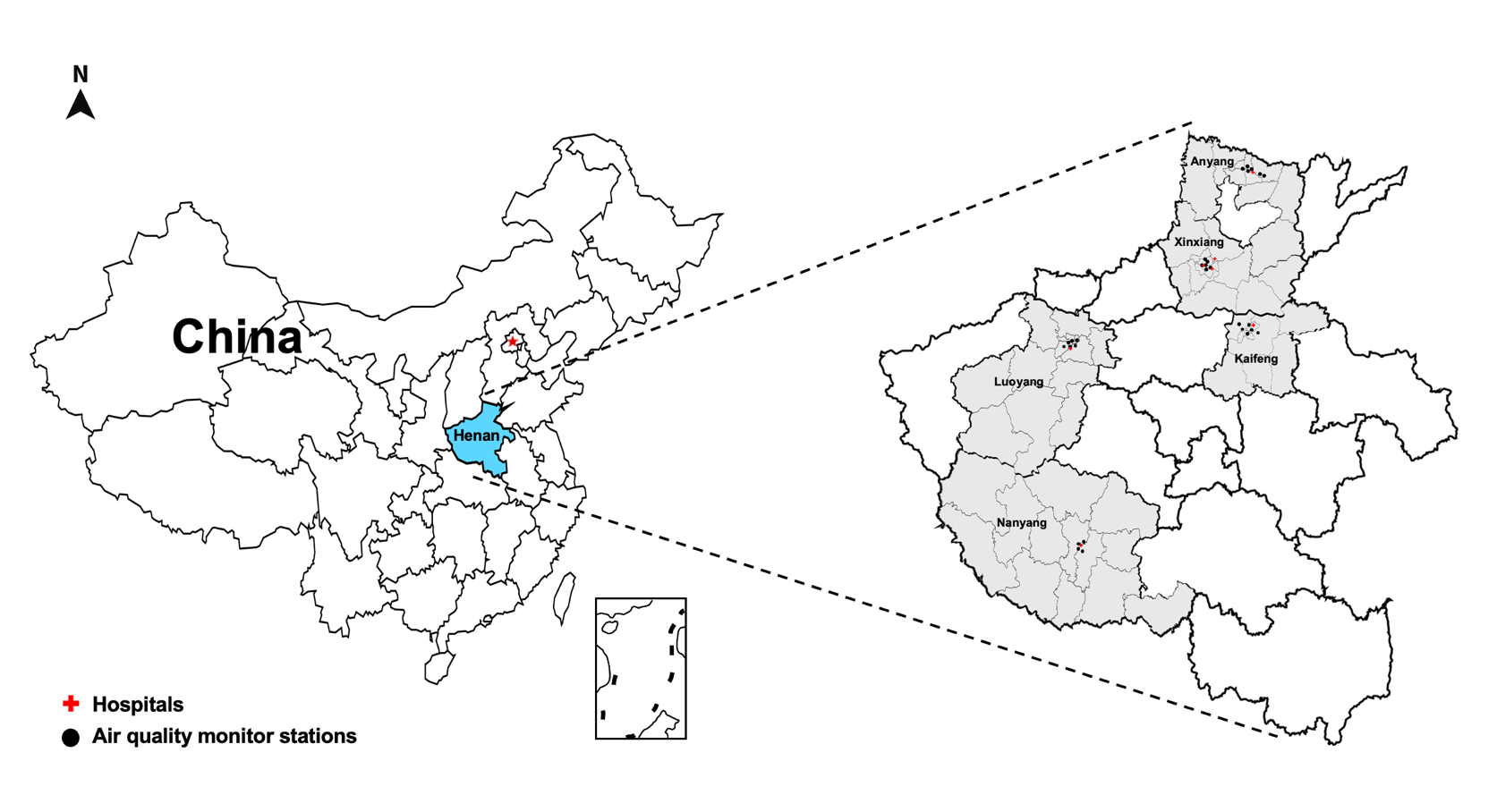


**Supplementary Figure 2. Percentage of O_3_ concentration interval in Henan Province during the study period (A) and average O_3_ concentration per month from 2016-2021 in different cities (B)**


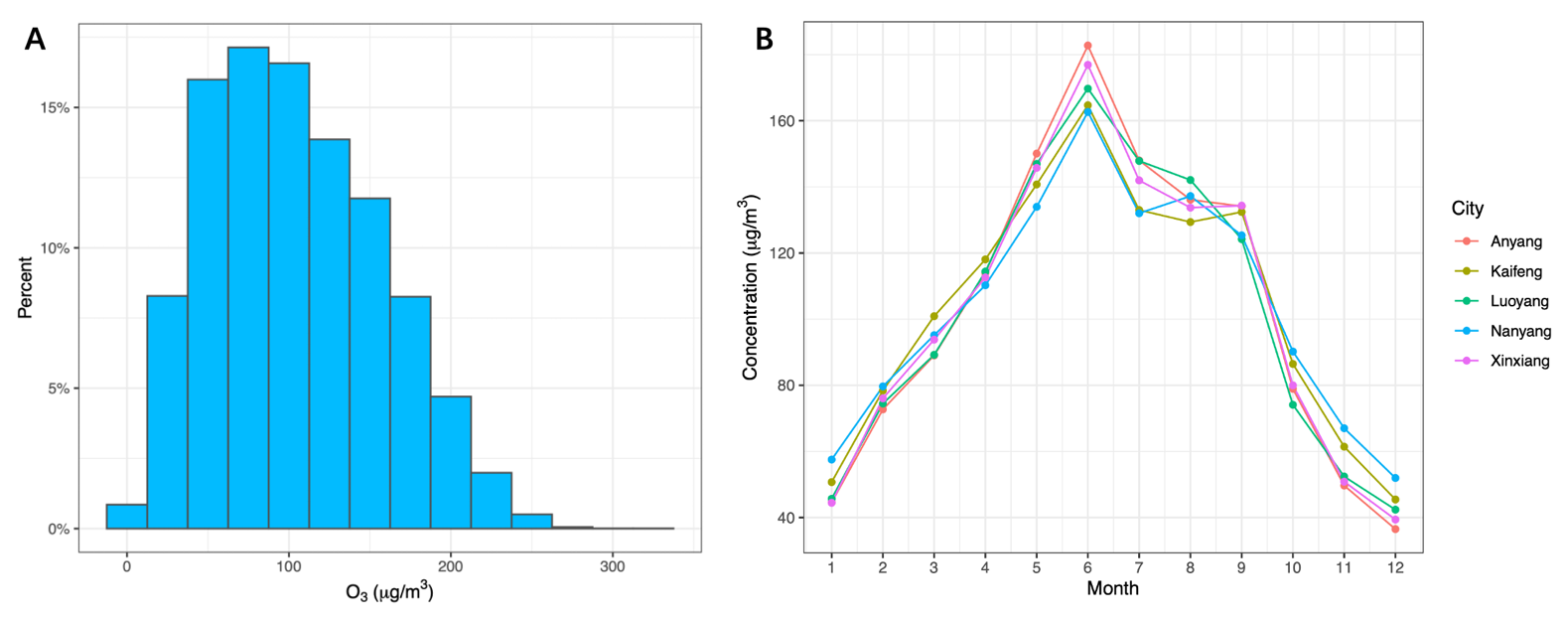


Note: O_3_: ozone.

**Supplementary Figure 3. Association between O_3_ single-/dual-pollution model (every 10 µg/m^3^ increase) and AMI hospitalization at single-day lag**

**
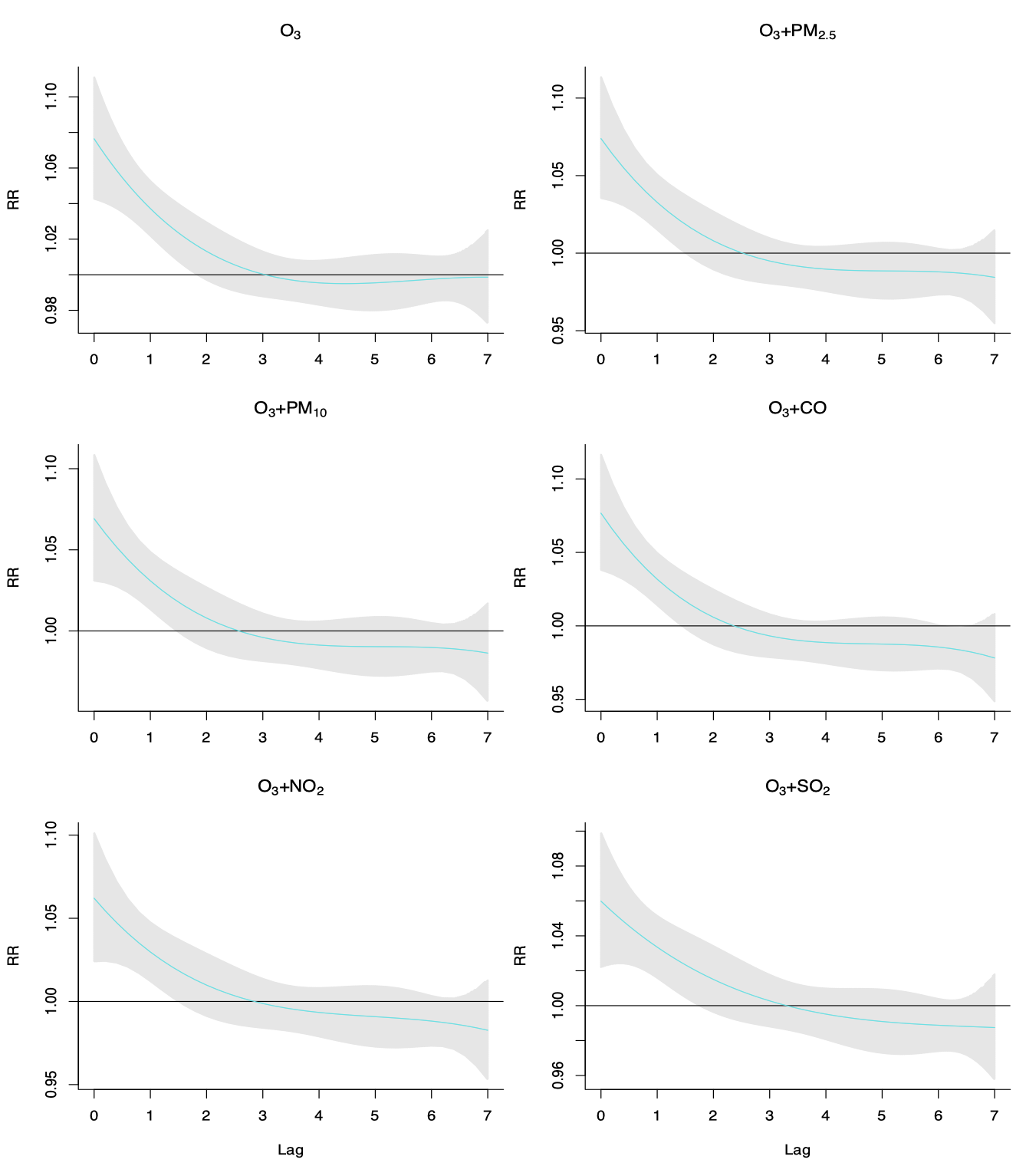
**

Note: PM_2.5_: particulate matter with aerodynamic matter ≤2.5 μm; PM_10_: particulate matter with aerodynamic matter ≤10 μm; CO: carbon monoxide; NO_2_ : nitrogen dioxide; SO_2_ : sulfur dioxide; O_3_ : ozone; Gray area: 95% confidence interval.


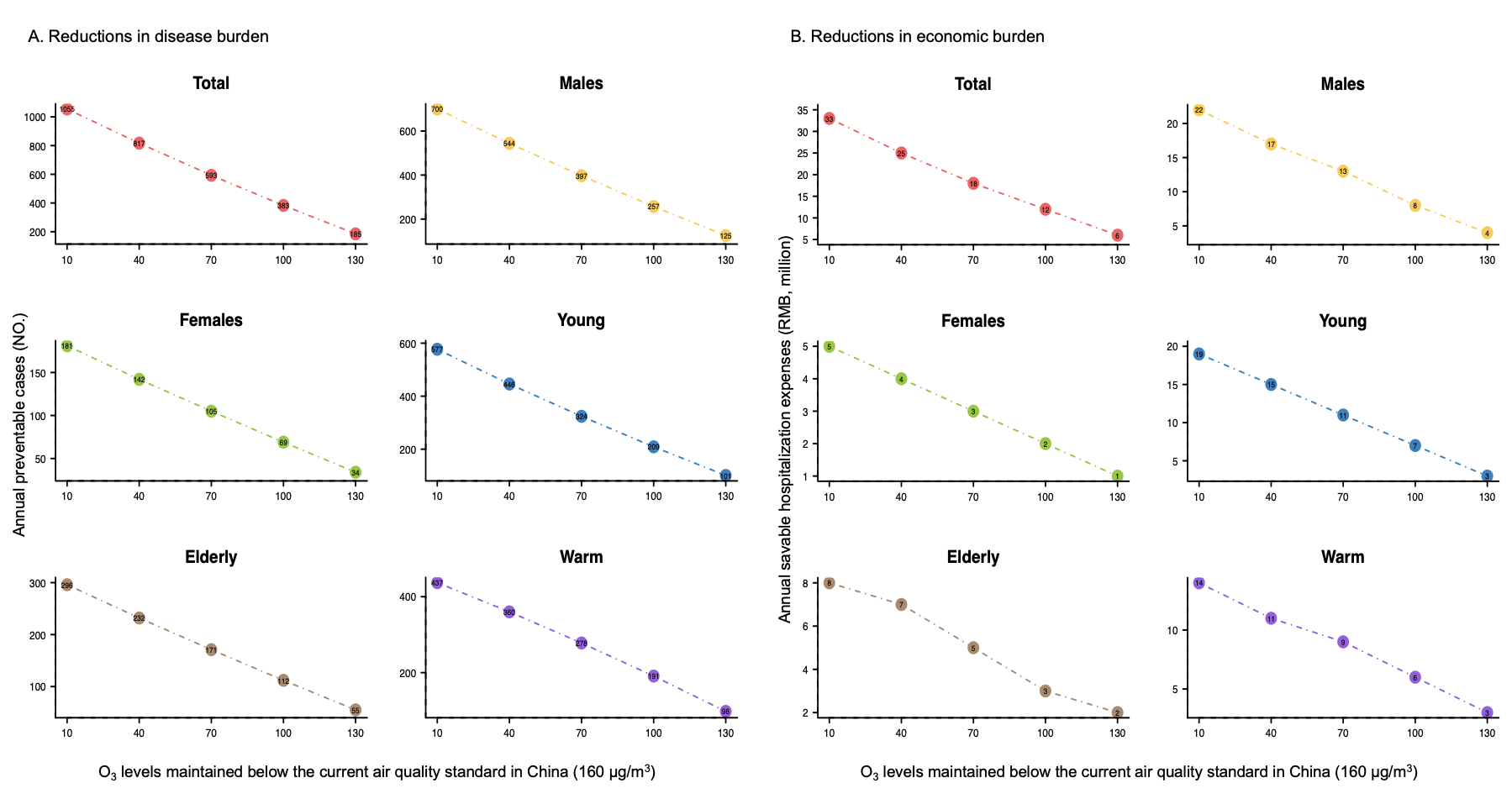
**Supplementary Figure 4. The estimated annual preventable AMI hospitalizations (A) and annual savable hospitalization costs (B) if the historical O_3_ concentration could be reduced to a relatively low level**

Note: young: 15–64 years; elderly: 65+ years; Warm: May to September.

**Supplementary Table 1. Coordinates of each hospital in the study area**

| City | Hospital Name | Longitude, N | Latitude, E |
| --- | --- | --- | --- |
| Xinxiang | The First Affiliated Hospital of Xinxiang Medical University | 114.059 | 35.408 |
|  | The Third Affiliated Hospital of Xinxiang Medical University | 113.925 | 35.281 |
|  | The Affiliated People's Hospital of Xinxiang Medical University | 113.869 | 35.306 |
| Nanyang | Nanyang Central Hospital | 112.529 | 33.008 |
| Anyang | Anyang First People's Hospital | 114.412 | 36.076 |
| Kaifeng | Hospital of Traditional Chinese Medicine Affiliated to Henan University | 114.368 | 34.805 |
| Luoyang | The First Affiliated Hospital of Henan University of Science and Technology | 112.427 | 34.599 |

**Supplementary Table 2. Coordinates of each city air monitoring station in the study area**

| City | City code | Station code | Longitude, N | Latitude, E |
| --- | --- | --- | --- | --- |
| Xinxiang | 410700 | 2389A | 113.836 | 35.310 |
|  |  | 2390A | 113.884 | 35.272 |
|  |  | 2391A | 113.883 | 35.303 |
|  |  | 3054A | 113.919 | 35.285 |
|  |  | 3476A | 113.914 | 35.333 |
|  |  | 3689A | 113.900 | 35.372 |
| Nanyang | 411300 | 2403A | 112.519 | 32.992 |
|  |  | 2404A | 112.522 | 33.012 |
|  |  | 2406A | 112.557 | 33.027 |
|  |  | 2407A | 112.550 | 32.968 |
| Anyang | 410500 | 1818A | 114.484 | 36.062 |
|  |  | 1819A | 114.355 | 36.102 |
|  |  | 1820A | 114.358 | 36.087 |
|  |  | 3141A | 114.392 | 36.088 |
|  |  | 3669A | 114.471 | 36.064 |
|  |  | 3703A | 114.292 | 36.095 |
| Kaifeng | 410200 | 1824A | 114.339 | 34.778 |
|  |  | 1826A | 114.289 | 34.797 |
|  |  | 3147A | 114.344 | 34.811 |
|  |  | 3210A | 114.364 | 34.796 |
|  |  | 3473A | 114.249 | 34.831 |
|  |  | 3592A | 114.464 | 34.756 |
| Luoyang | 410300 | 1812A | 112.394 | 34.651 |
|  |  | 1814A | 112.466 | 34.687 |
|  |  | 1815A | 112.494 | 34.689 |
|  |  | 1817A | 112.428 | 34.626 |
|  |  | 3341A | 112.495 | 34.633 |
|  |  | 3636A | 112.439 | 34.678 |

**Supplementary Table 3. Spearman’s correlation coefficients between daily air pollutants (O_3_: 8h) and meteorological data in Henan, 2016–2021**

|  | PM_2.5_ | PM_10_ | CO | NO_2_ | SO_2_ | O_3_ | Temperature | Sunshine Duration |
| --- | --- | --- | --- | --- | --- | --- | --- | --- |
| PM_2.5_ | 1.00^*^ |  |  |  |  |  |  |  |
| PM_10_ | 0.88^*^ | 1.00^*^ |  |  |  |  |  |  |
| CO | 0.73^*^ | 0.68^*^ | 1.00^*^ |  |  |  |  |  |
| NO_2_ | 0.67^*^ | 0.69^*^ | 0.65^*^ | 1.00^*^ |  |  |  |  |
| SO_2_ | 0.46^*^ | 0.53^*^ | 0.67^*^ | 0.60^*^ | 1.00^*^ |  |  |  |
| O_3_ | -0.42^*^ | -0.33^*^ | -0.35^*^ | -0.43^*^ | -0.21^*^ | 1.00^*^ |  |  |
| Temperature | -0.52^*^ | -0.43^*^ | -0.40^*^ | -0.49^*^ | -0.32^*^ | 0.78^*^ | 1.00^*^ |  |
| Sunshine Duration | -0.26^*^ | -0.12^*^ | -0.21^*^ | -0.09^*^ | 0.03^*^ | 0.54^*^ | 0.34^*^ | 1.00^*^ |

Note: PM_2.5_ : particulate matter with aerodynamic matter ≤2.5 μm; PM_10_ : particulate matter with aerodynamic matter ≤10 μm; CO : carbon monoxide; NO_2_ : nitrogen dioxide; SO_2_ : sulfur dioxide; O_3_ : ozone; ^*^：p<0.001.

**Supplementary Table 4. RR (95% CI) of AMI hospitalizations with an increase of 10μg/m^3^ in air pollutants (and 1mg/m^3^ in CO) according to the single-pollutant model at different lag days.**

|  | O_3_ | | PM_2.5_ | | PM_10_ | | CO | | NO_2_ | | SO_2_ | |
| --- | --- | --- | --- | --- | --- | --- | --- | --- | --- | --- | --- | --- |
|  | RR | 95%CI | RR | 95%CI | RR | 95%CI | RR | 95%CI | RR | 95%CI | RR | 95%CI |
| Lag0 | **1.076** | **(1.043-1.111)** | **1.017** | **(1.003-1.031)** | **1.017** | **(1.005-1.028)** | 1.029 | (0.983-1.078) | 1.034 | (0.986-1.085) | 0.993 | (0.973-1.014) |
| Lag1 | **1.037** | **(1.022-1.053)** | **1.011** | **(1.005-1.017)** | **1.009** | **(1.004-1.015)** | **1.020** | **(1.000-1.041)** | **1.029** | **(1.008-1.051)** | 0.994 | (0.984-1.003) |
| Lag2 | 1.013 | (0.997-1.029) | 1.005 | (0.998-1.012) | 1.004 | (0.997-1.010) | 1.007 | (0.982-1.032) | 1.019 | (0.993-1.045) | 0.994 | (0.983-1.005) |
| Lag3 | 1.000 | (0.988-1.013) | 1.000 | (0.995-1.006) | 1.000 | (0.995-1.005) | 0.993 | (0.973-1.012) | 1.005 | (0.985-1.025) | 0.994 | (0.985-1.003) |
| Lag4 | 0.995 | (0.983-1.008) | 0.997 | (0.991-1.002) | 0.998 | (0.993-1.002) | 0.982 | (0.963-1.001) | 0.990 | (0.971-1.010) | 0.993 | (0.984-1.001) |
| Lag5 | 0.995 | (0.980-1.011) | 0.995 | (0.988-1.002) | 0.997 | (0.991-1.004) | 0.977 | (0.953-1.002) | 0.979 | (0.954-1.004) | 0.989 | (0.978-1.000) |
| Lag6 | 0.997 | (0.985-1.01) | 0.997 | (0.991-1.003) | 0.999 | (0.994-1.005) | 0.983 | (0.963-1.003) | 0.972 | (0.952-0.993) | 0.982 | (0.974-0.991) |
| Lag7 | 0.999 | (0.973-1.025) | 1.002 | (0.990-1.015) | 1.004 | (0.993-1.015) | 1.002 | (0.960-1.045) | 0.973 | (0.933-1.016) | 0.972 | (0.954-0.991) |
| Lag01 | **1.116** | **(1.073-1.161)** | **1.028** | **(1.011-1.045)** | **1.026** | **(1.012-1.040)** | 1.050 | (0.996-1.107) | **1.065** | **(1.007-1.126)** | 0.987 | (0.963-1.011) |
| Lag02 | **1.131** | **(1.085-1.179)** | **1.033** | **(1.016-1.051)** | **1.030** | **(1.015-1.045)** | **1.057** | **(1.003-1.115)** | **1.085** | **(1.025-1.148)** | 0.981 | (0.957-1.006) |
| Lag03 | **1.132** | **(1.083-1.182)** | **1.033** | **(1.015-1.052)** | **1.029** | **(1.014-1.045)** | 1.049 | (0.993-1.110) | **1.089** | **(1.027-1.156)** | 0.975 | (0.950-1.001) |
| Lag04 | **1.126** | **(1.077-1.178)** | **1.030** | **(1.011-1.049)** | **1.027** | **(1.010-1.044)** | 1.030 | (0.973-1.091) | **1.079** | **(1.015-1.147)** | 0.968 | (0.942-0.995) |
| Lag05 | **1.121** | **(1.070-1.175)** | **1.025** | **(1.004-1.047)** | **1.024** | **(1.006-1.043)** | 1.007 | (0.945-1.073) | 1.056 | (0.988-1.128) | 0.958 | (0.931-0.985) |
| Lag06 | **1.118** | **(1.064-1.176)** | 1.022 | (0.999-1.046) | **1.024** | **(1.004-1.044)** | 0.989 | (0.924-1.059) | 1.026 | (0.957-1.101) | 0.941 | (0.914-0.969) |
| Lag07 | **1.117** | **(1.064-1.172)** | **1.024** | **(1.001-1.048)** | **1.028** | **(1.007-1.049)** | 0.991 | (0.930-1.055) | 0.999 | (0.934-1.069) | 0.914 | (0.891-0.938) |

Note: O_3_: ozone; PM_2.5_: particulate matter with aerodynamic matter ≤2.5 μm; PM_10_: particulate matter with aerodynamic matter ≤10 μm; CO: carbon monoxide; NO_2_: nitrogen dioxide; SO_2_: sulfur dioxide; Bold: p<0.05.

**Supplementary Table 5. RR (95% CI) of AMI hospitalizations at different lag days with O_3_ (every 10 µg/m^3^ increase) in different subgroups.**

|  | Males | Females | Young | Elderly | Warm | Cold | LSD | SSD |
| --- | --- | --- | --- | --- | --- | --- | --- | --- |
|  | RR (95%CI) | RR (95%CI) | RR (95%CI) | RR (95%CI) | RR (95%CI) | RR (95%CI) | RR (95%CI) | RR (95%CI) |
| Lag0 | **1.073 (1.031-1.117)** | **1.066 (1.010-1.126)** | **1.094 (1.052-1.139)** | **1.054 (1.010-1.101)** | **1.095 (1.049-1.143)** | 1.069 (0.989-1.156) | **1.144 (1.057-1.237)** | 1.060 (0.989-1.136) |
| Lag1 | **1.034 (1.014-1.054)** | **1.029 (1.003-1.055)** | **1.042 (1.023-1.062)** | **1.031 (1.010-1.052)** | **1.053 (1.033-1.072)** | 1.027 (0.991-1.065) | **1.046 (1.007-1.085)** | 1.028 (0.994-1.064) |
| Lag2 | 1.010 (0.989-1.031) | 1.006 (0.979-1.034) | 1.011 (0.992-1.032) | 1.015 (0.993-1.037) | 1.025 (1.005-1.044) | 0.998 (0.963-1.035) | 1.004 (0.967-1.043) | 1.015 (0.977-1.054) |
| Lag3 | 0.997 (0.981-1.013) | 0.995 (0.973-1.017) | 0.997 (0.981-1.013) | 1.005 (0.988-1.022) | 1.007 (0.992-1.023) | 0.981 (0.936-1.028) | 0.996 (0.965-1.027) | 1.013 (0.982-1.044) |
| Lag4 | 0.992 (0.976-1.008) | 0.992 (0.970-1.013) | 0.993 (0.978-1.009) | 0.999 (0.982-1.016) | 0.999 (0.983-1.014) | 0.976 (0.932-1.021) | 1.003 (0.972-1.034) | 1.015 (0.984-1.047) |
| Lag5 | 0.990 (0.970-1.010) | 0.994 (0.968-1.022) | 0.996 (0.977-1.016) | 0.994 (0.973-1.016) | 0.995 (0.976-1.015) | 0.982 (0.950-1.014) | 1.007 (0.97-1.045) | 1.015 (0.977-1.055) |
| Lag6 | 0.988 (0.972-1.005) | 1.000 (0.978-1.023) | 1.003 (0.987-1.019) | 0.991 (0.974-1.008) | 0.995 (0.979-1.011) | 0.999 (0.964-1.035) | 0.992 (0.96-1.026) | 1.007 (0.974-1.040) |
| Lag7 | 0.984 (0.951-1.018) | 1.007 (0.962-1.054) | 1.009 (0.976-1.043) | 0.986 (0.952-1.022) | 0.996 (0.965-1.029) | 1.028 (0.947-1.116) | 0.943 (0.887-1.002) | 0.982 (0.923-1.046) |
| Lag01 | **1.109 (1.056-1.166)** | **1.097 (1.026-1.174)** | **1.141 (1.086-1.198)** | **1.087 (1.031-1.146)** | **1.153 (1.093-1.216)** | 1.099 (0.986-1.224) | **1.196 (1.081-1.322)** | **1.090 (1.001-1.187)** |
| Lag02 | **1.120 (1.063-1.181)** | **1.104 (1.028-1.185)** | **1.154 (1.095-1.215)** | **1.103 (1.043-1.167)** | **1.181 (1.117-1.248)** | 1.097 (0.979-1.228) | **1.201 (1.077-1.339)** | **1.107 (1.009-1.213)** |
| Lag03 | **1.117 (1.057-1.180)** | **1.098 (1.019-1.183)** | **1.150 (1.089-1.214)** | **1.109 (1.045-1.176)** | **1.190 (1.124-1.259)** | 1.076 (0.957-1.209) | **1.196 (1.065-1.342)** | **1.121 (1.015-1.237)** |
| Lag04 | **1.108 (1.047-1.172)** | **1.088 (1.008-1.175)** | **1.142 (1.080-1.207)** | **1.107 (1.042-1.176)** | **1.188 (1.122-1.257)** | 1.050 (0.925-1.192) | **1.199 (1.063-1.353)** | **1.138 (1.026-1.261)** |
| Lag05 | **1.096 (1.033-1.163)** | 1.082 (0.998-1.174) | **1.138 (1.072-1.207)** | **1.101 (1.033-1.174)** | **1.182 (1.113-1.256)** | 1.031 (0.902-1.178) | **1.207 (1.061-1.374)** | **1.155 (1.034-1.291)** |
| Lag06 | **1.084 (1.018-1.153)** | 1.083 (0.994-1.179) | **1.141 (1.072-1.214)** | **1.091 (1.020-1.167)** | **1.176 (1.104-1.254)** | 1.029 (0.911-1.163) | **1.198 (1.045-1.374)** | **1.163 (1.033-1.309)** |
| Lag07 | **1.066 (1.004-1.132)** | **1.090 (1.004-1.184)** | **1.151 (1.084-1.223)** | **1.076 (1.008-1.149)** | **1.172 (1.102-1.247)** | 1.058 (0.946-1.183) | 1.13 (0.986-1.295) | **1.142 (1.015-1.286)** |

Note: Warm: May to September; Cold: from November to March; LSD: Long Sunshine Duration (sunshine duration > 6h); SSD: Short Sunshine Duration (sunshine duration ≤ 6h); Both LSD and SSD are warm season sunshine durations; Bold: p<0.05.

**Supplementary Table 6. Comparison of the AMI hospitalizations, O_3_ concentration and mean temperature in cold and warm seasons**

|  | Mean ± SD | Min | 25^th^ | 50^th^ | 75^th^ | Max |
| --- | --- | --- | --- | --- | --- | --- |
| Cold | | | | | | |
| AMI | 12.01±4.79 | 1.00 | 9.00 | 11.00 | 14.00 | 36.00 |
| O_3_ (µg/m^3^) | 56.15±26.06 | 4.00 | 37.00 | 54.00 | 72.00 | 160.00 |
| Mean temperature (°C) | 4.60±4.77 | -10.20 | 1.20 | 4.10 | 7.60 | 19.60 |
| Sunshine duration (h) | 4.62±3.32 | 0.00 | 1.12 | 5.04 | 7.64 | 10.88 |
| Warm | | | | | | |
| AMI | 11.07±4.39 | 1.00 | 8.00 | 11.00 | 14.00 | 31.00 |
| O_3_ (µg/m^3^) | 143.77±43.56 | 26.00 | 112.00 | 144.00 | 174.00 | 316.00 |
| Mean temperature (°C) | 25.26±3.65 | 13.10 | 22.80 | 25.70 | 28.00 | 34.60 |
| Sunshine duration (h) | 6.33±3.66 | 0.00 | 3.27 | 6.72 | 9.64 | 13.18 |

Note: Warm: May to September; Cold: from November to March (next year); O_3_: ozone.

**Supplementary Table 7. Comparison of the AMI hospitalizations, O_3_ concentration, average temperature and sunshine duration in warm seasons**

|  | Mean ± SD | Min | 25^th^ | 50^th^ | 75^th^ | Max |
| --- | --- | --- | --- | --- | --- | --- |
| LSD | | | | | | |
| AMI | 10.24±3.73 | 2.00 | 8.00 | 10.00 | 12.00 | 24.00 |
| O_3_ (µg/m^3^) | 165.20±36.40 | 64.00 | 140.00 | 165.00 | 190.00 | 316.00 |
| Average temperature (℃) | 26.53±3.28 | 16.20 | 24.40 | 26.90 | 28.90 | 34.60 |
| Sunshine duration (h) | 9.52±1.78 | 6.10 | 8.00 | 9.70 | 11.00 | 13.50 |
| SSD | | | | | | |
| AMI | 9.93±3.86 | 1.00 | 7.00 | 10.00 | 12.75 | 24.00 |
| O_3_ (µg/m^3^) | 120.20±37.78 | 26.00 | 93.00 | 120.00 | 146.00 | 253.00 |
| Average temperature (℃) | 23.83±3.56 | 13.10 | 21.40 | 24.30 | 26.43 | 33.90 |
| Sunshine duration (h) | 2.37±1.95 | 0.00 | 0.50 | 2.10 | 4.10 | 6.00 |

Note: LSD: Long Sunshine Duration (sunshine duration > 6h); SSD: Short Sunshine Duration (sunshine duration ≤ 6h); O_3_: ozone.

**Supplementary Table 8. Attributable fraction of hospitalizations to single- and dual-pollution models of O_3_ exposure (%)**

|  | O_3_ | O_3_+PM_2.5_ | O_3_+PM_10_ | O_3_+CO | O_3_+NO_2_ | O_3_+SO_2_ |
| --- | --- | --- | --- | --- | --- | --- |
| Total | 11.66 (7.66-15.40) | 10.07 (5.48-14.46) | 9.67 (5.03-14.02) | 9.91 (5.30-14.31) | 9.34 (4.67-13.79) | 10.31 (5.75-14.68) |
| Males | 10.47 (5.39-15.25) | 10.8 (5.73-15.59) | 10.54 (5.47-15.34) | 10.78 (5.71-15.57) | 10.42 (5.33-15.24) | 11.36 (6.31-16.14) |
| Females | 8.93 (1.86-15.47) | 8.25 (0.77-15.16) | 7.21 (-0.35-14.19) | 7.52 (0.00-14.48) | 6.41 (-1.23-13.46) | 7.53 (0.00-14.49) |
| Young | 13.04 (8.17-17.63) | 12.41 (6.87-17.63) | 11.98 (6.41-17.21) | 12.23 (6.68-17.46) | 11.53 (5.93-16.80) | 12.91 (7.39-18.10) |
| Elderly | 9.83 (4.31-14.97) | 7.33 (1.24-13.04) | 6.83 (0.71-12.57) | 7.08 (0.97-12.81) | 6.75 (0.61-12.51) | 7.27 (1.16-13.00) |

Note: Young: 15–64 years; Elderly: 65+ years; O_3_: ozone; PM_2.5_: particulate matter with aerodynamic matter ≤2.5 μm; PM_10_: particulate matter with aerodynamic matter ≤10 μm; CO: carbon monoxide; NO_2_: nitrogen dioxide; SO_2_: sulfur dioxide.

**Supplementary Table 9. Results of sensitivity analyses by changing degree of freedom for long-term trend and seasonality**

|  | Temp | Total | Males | Females | Young | Elderly | Warm | Cold |
| --- | --- | --- | --- | --- | --- | --- | --- | --- |
| df=3 | 5 | 1.121(1.073-1.172) | 1.128(1.074-1.185) | 1.102(1.021-1.189) | 1.137(1.075-1.201) | 1.102(1.037-1.170) | 1.183(1.118-1.253) | 1.117(0.989-1.261) |
| df=4 | 5 | 1.128(1.079-1.180) | 1.132(1.078-1.190) | 1.117(1.034-1.206) | 1.142(1.080-1.207) | 1.111(1.046-1.180) | 1.188(1.120-1.260) | 1.120(0.988-1.268) |
| df=5 | 5 | 1.103(1.082-1.185) | 1.139(1.083-1.196) | 1.115(1.032-1.205) | 1.149(1.086-1.216) | 1.111(1.045-1.181) | 1.191(1.122-1.264) | 1.118(0.986-1.268) |
| df=6 | 5 | 1.103(1.080-1.184) | 1.139(1.083-1.198) | 1.108(1.025-1.199) | 1.154(1.090-1.222) | 1.102(1.036-1.173) | 1.194(1.123-1.269) | 1.122(0.989-1.274) |
| df=7 | 5 | 1.103(1.079-1.183) | 1.138(1.081-1.197) | 1.108(1.024-1.200) | 1.147(1.082-1.215) | 1.108(1.041-1.179) | 1.197(1.126-1.272) | 1.124(0.989-1.277) |

Note: Df: degree of freedom; Young: 15–64 years; Elderly: 65+ years; Warm: May to September; Cold: from November to March (next year).
